# Sounding-rocket microgravity experiments on alumina dust

**DOI:** 10.1038/s41467-018-06359-y

**Published:** 2018-09-19

**Authors:** Shinnosuke Ishizuka, Yuki Kimura, Itsuki Sakon, Hiroshi Kimura, Tomoya Yamazaki, Shinsuke Takeuchi, Yuko Inatomi

**Affiliations:** 1 Institute of Low Temperature Science, Hokkaido University, Hokkaido Sapporo, 060-0819 Japan; 2 Department of Astronomy, School of Science, University of Tokyo, 7-3-1 Hongo, Bunkyo-ku, Tokyo, 113-0033 Japan; 3 Planetary Exploration Research Center (PERC), Chiba Institute of Technology, Tsudanuma 2-17-1, Narashino Chiba, 275-0016 Japan; 4 Institute of Space and Astronautical Science, Japan Aerospace Exploration Agency, 3-1-1 Yoshinodai, Chuo-ku, Kanagawa Sagamihara, 229-8510 Japan; 5 School of Physical Sciences, SOKENDAI (The Graduate University for Advanced Studies), 3-1-1 Yoshinodai, Chuo-ku, Kanagawa Sagamihara, 252-5210 Japan

## Abstract

Alumina (Al_2_O_3_) is believed to be the first major condensate to form in the gas outflow from oxygen-rich evolved stars because of the refractoriness and that α-Al_2_O_3_ (corundum, most stable polymorph) is a potential origin of a 13 μm feature that appears close to stars. However, no one has directly reproduced the 13 μm feature experimentally, and it has remained as a noteworthy unidentified infrared band. Here, we report nucleation experiments on Al_2_O_3_ nanoparticles monitored by a specially designed infrared spectrometer in the microgravity environment of a sounding rocket. The conditions approximate to those around asymptotic giant branch (AGB) stars. The measured spectra of the nucleated Al_2_O_3_ show a sharp feature at a wavelength of 13.55 μm and comparable in width to that observed near oxygen-rich AGB stars. Our finding that α-Al_2_O_3_ nucleates under certain condition provides a solid basis to elaborate condensation models of dust around oxygen-rich evolved stars.

## Abstract (web summary)

Alumina is thought to be the main condensate to form in the gas outflow from oxygen-rich evolved stars. Here, the authors perform a condensation experiment with alumina in a low-gravity environment, and find spectroscopic evidence for a sharp feature at a wavelength of 13.55 μm.

## Introduction

Asymptotic giant branch (AGB) stars, a type of evolved stars, are significant sources of the dust present in interstellar space. Advances in interferometric observations of low-mass stars, such as AGB stars in their late evolution stages, have demonstrated the existence of shells consisting of relatively large (~300 nm) grains close (within two stellar radii) to the stars^1^. Therefore, the dust must be refractory to condense, and abundant to be detected by observations. Recently, the Atacama Large Millimeter Array (ALMA) visualized the spatial distribution of molecular rotational bands around the alumina (Al_2_O_3_)-rich AGB star W Hya demonstrating that AlO gases are consumed within the dust formation region while SiO molecules remain gaseous even at the outside of the dust formation region^2^. Further, the presence of titania (TiO_2_) in the gas phase far from the dust shell is reported for a red supergiant environment^3^. Although several minerals, such as Al_2_O_3_, TiO_2_, and spinel (MgAl_2_O_4_), have been proposed as carriers of the 13 μm feature^4^, Al_2_O_3_ is the most likely mineral to be capable of surviving sublimation and, owing to its transparency, of withstanding the radiation pressure from the central star; furthermore, it is considered to condense earlier than other abundant minerals, such as silicates. The discovery of micron-sized presolar Al_2_O_3_ dust with an unusual oxygen isotopic composition in meteorites strongly suggests that some of the Al_2_O_3_ dust has been formed around AGB stars. e.g. ref. ^5^.

Alumina exhibits a number of crystalline structures (e.g., α, θ, δ, or γ); i.e., it can exist in a diversity of polymorphic forms. Significant differences in the oxygen anion alignment in hexagonal closed-packed α-Al_2_O_3_ (corundum) and in face-centered cubic γ-, δ-, or θ-Al_2_O_3_ lead to distinct differences in the corresponding infrared (IR) band structures^6^. Amorphous and γ-Al_2_O_3_ have been considered to contribute to the 10–20 μm dust extinction around red supergiant stars and AGB stars^7–9^. In addition, α-Al_2_O_3_, the form of Al_2_O_3_ that is stable from 0 K to the melting point in the bulk size, has been proposed as a candidate for the origin of the unidentified 13 μm feature that has been observed for AGB stars with low mass-loss rates^10^. The IR features in the spectrum of mineral particles have been calculated by using optical constants obtained for the corresponding large single crystals, based on Mie theories. The features vary with the size distribution^11^, temperature^12^, and shape formed in anisotropic crystal growth^13^. The spectral profiles have been successfully reproduced by Al_2_O_3_ with an appropriate choice of these parameters.

However, the 13 μm feature has not been reproduced experimentally. The average feature observed for the unidentified 13 μm band has a full width at half maximum (FWHM) of 0.5–0.8^13^, 0.6^14^, or 1.1 μm^15^. In contrast to the narrow feature observed astronomically, spectra measured on powders prepared in the laboratory have shown a markedly greater width (Δ*λ* = 6.0 μm). This is also true for measurements of the IR spectrum of α-Al_2_O_3_ particles in a free-flying state in a medium with a dielectric constant that approximates to that of the astronomical environment^16^. In addition to the size distribution, shape, and anisotropy, the electromagnetic interactions between particles induced during sample preparation broaden the peak^17,18^. These effects prevent a direct comparison with astronomical observations.

We performed in situ IR measurements on free-flying Al_2_O_3_ in condensation experiments involving homogeneous nucleation in a ground-based laboratory; an IR spectrum of monodispersed Al_2_O_3_ particles of uniform size and shape was successfully obtained^19^. However, an α-Al_2_O_3_ phase has never been directly synthesized by nucleation and growth processes in the vapor phase^19^. We believe the reason for this is the rapid cooling of the nascent Al_2_O_3_ particles by the thermal convection of the argon (Ar) buffer gas used to reduce the mean free path of the evaporated vapor, and thereby to reduce the physical scale of the experimental system. In the ground-based experiment, a strong convection (10–15 cm s^−1^) of Ar gas is induced by the hot evaporation source. By contrast, in a microgravitational environment, this thermal convection is completely suppressed, allowing the evaporated gases to cool more slowly. Consequently, the Al_2_O_3_ nuclei have higher possibility to overcome a large barrier to form the most stable corundum phase. In addition, the slower cooling permits the gaseous atoms to collide more frequently and, on a longer timescale of gas cooling, provides a more realistic simulation of the grain formation in gas outflows from AGB stars^20^. Based on this concept, we performed an ideal-nucleation experiment in a microgravitational environment of 2.2 × 10^−3^ m s^−2^ (2.2 × 10^−4^ G) aboard the sounding rocket S-520 30 and successfully reproduce a 13 μm band with a very narrow width of 0.5–0.6 μm. The experimental method will provide sufficient data to permit the identification of the unidentified stellar IR bands.

## Results

### Formation of Al_2_O_3_ in a microgravitational environment

**Fig. 1:**
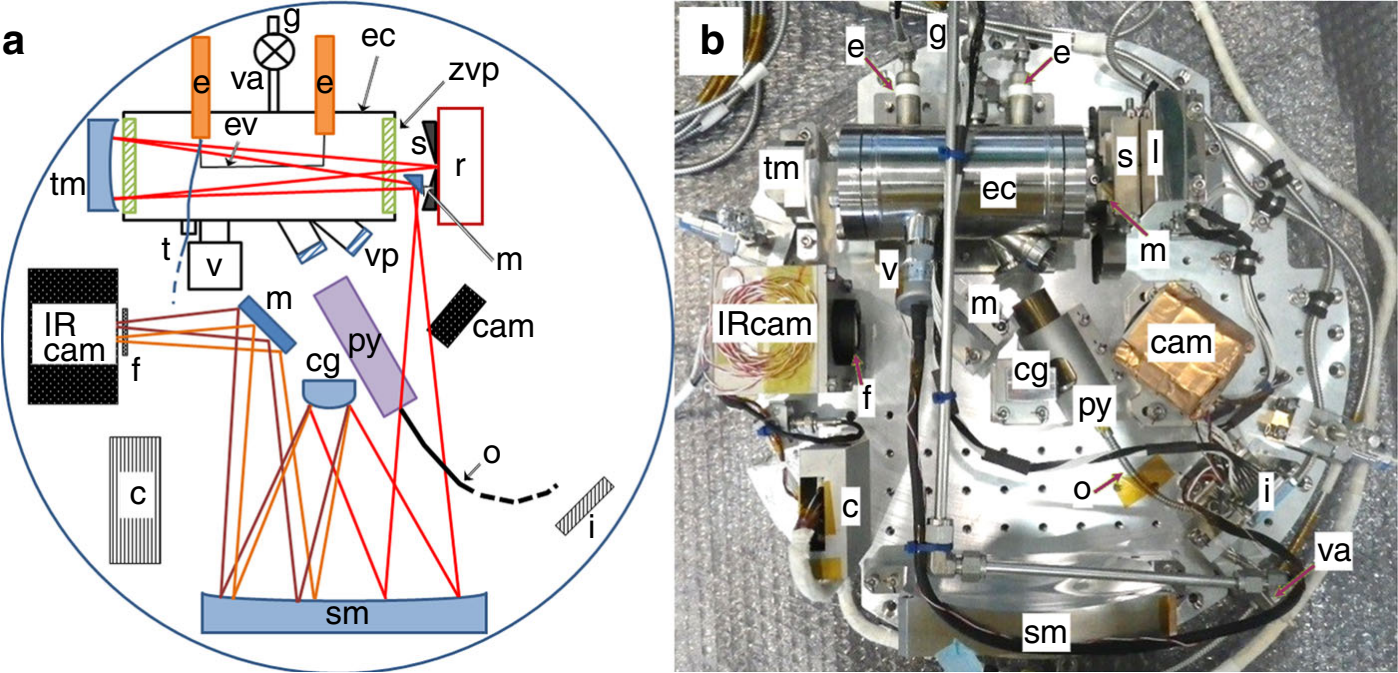
The optical system of the dispersive IR spectrometer with the experimental chamber. **a** Schematic representation; **b** corresponding photograph. The IR radiation (magenta lines) was dispersed at the convex grating (cg). The resulting intensity of the IR radiation was recorded with an IR camera (IR cam). The evaporation source (ev), consisting of an Al wire wrapped around a Ta filament (0.2 mm in diameter and 67 mm in length) is depicted by the black solid line in the experimental chamber (ec). The other labels are as follows: (c) IR camera controller; (e) electrode; (f) IR filter; (g) gas line; (i) interface connecters; (m) mirror; (o) optical fiber; (r) IR radiation source; (s) slit; (t) thermocouple; (v) vacuum gauge; (cam) CMOS camera; (py) pyrometer; (sm) spherical mirror; (tm) toroidal mirror; (va) valve; (vp) view port; (zvp) ZnSe view port. The evacuation of the air and subsequent injection of Ar gas into the chamber were performed on the ground after the experimental system had been installed inside the rocket

To measure the temporal evolution of the IR spectra of free-flying Al_2_O_3_ nanoparticles nucleated in the experimental chamber in a microgravitational environment, we have designed and fabricated an IR grating spectrometer system to meet the size limitations of the sounding rocket S-520, and endure impacts of the launch (Fig. 1 and Methods). The experimental chamber entered a microgravitational environment 50 s after the rocket launch, as confirmed by the acceleration measurements (Supplementary Fig. 1). Ninety seconds after the launch, the aluminum (Al) evaporation source was electrically heated on a tantalum (Ta) filament in a gas mixture of Ar (3.8 × 10^4^ Pa) and oxygen (2.0 × 10^3^ Pa). The evaporated Al reacted with the oxygen to form Al_2_O_3_ nanoparticles (Methods). The total pressure rose and then fell due to heating and cooling of the evaporation source, respectively (Supplementary Fig. 2). After the experiment, the total pressure was by 1700 Pa lower than before the experiment. Evidently, oxygen was consumed by the Al oxidation to form Al_2_O_3_ nanoparticles.

### Measurement of an infrared spectra with a narrow band

**Fig. 2:**
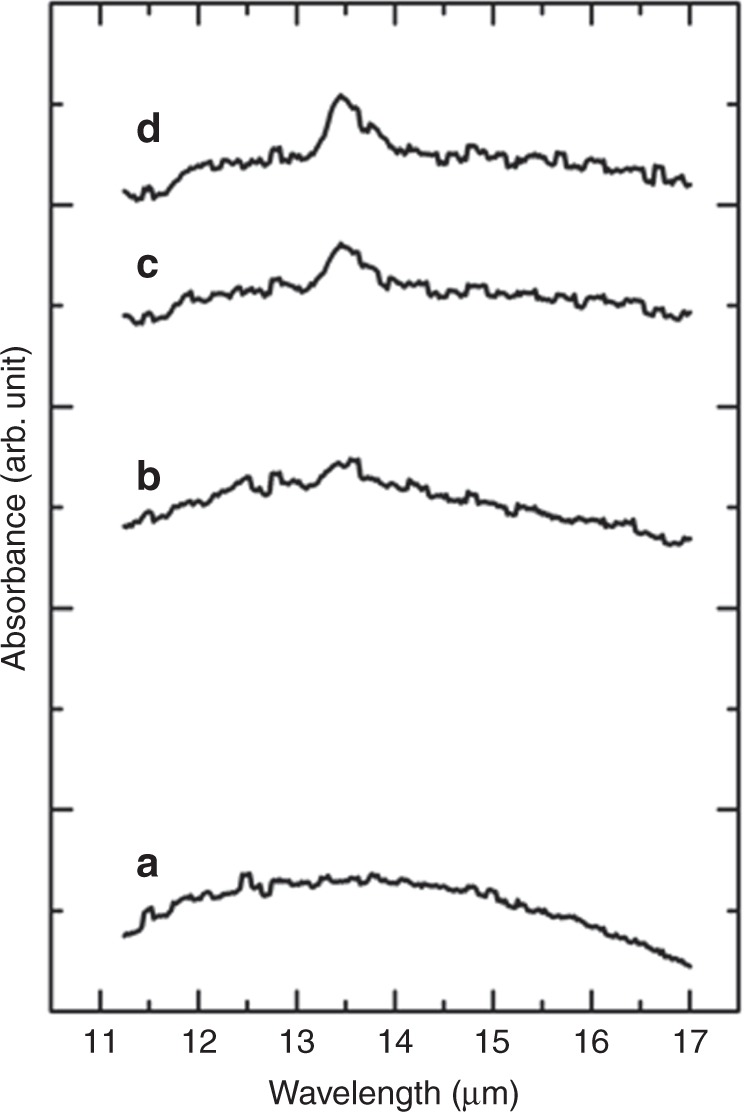
Absorption spectra of nascent Al_2_O_3_ nanoparticles. These spectra have been accumulated at (**a**) 97.6–100.2, (**b**) 103.0–104.5, and (**c**) 105.6–109.7 s after the launch; the background spectrum BG (Supplementary Fig. 3) was subtracted and the spectra were not offset. The 13.55 ± 0.05 μm absorbance that appeared in **c** remained for a long period. The stationary absorbance at 109.9–114.0 s after the launch is shown in **d**

**Fig. 3:**
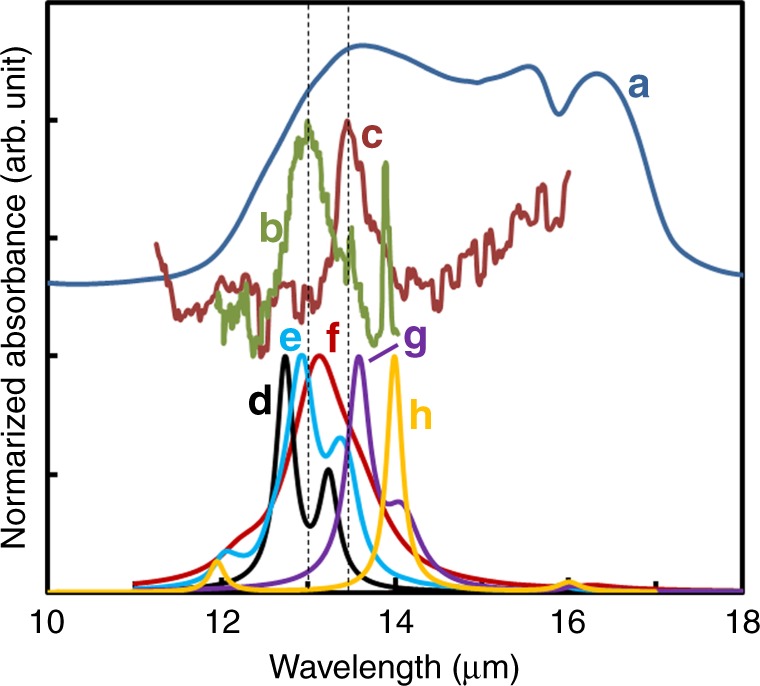
Comparison of IR spectra. These spectra were obtained: **a** with laboratory measurements on α-Al_2_O_3_ embedded in a KBr medium (blue)^23^; **b** by observation of AGB stars (khaki)^4^; **c** in this study (brown). Calculated spectra of **d**: spheres at 300 K (black); **e** faceted particle at 300 K (light blue); **f** spheres at 928 K (magenta); **g** spheres with surface contamination of Ta of 5 nm in thickness (purple); **h** oblate flattened along *c*-axis with ratio of 0.3 (yellow)

The raw spectra obtained for 10 Hz were divided into several series based on variations in the spectral shape during the experiment (Supplementary Fig. 3). We averaged 32 spectra recorded before the evaporation of Al_2_O_3_ in the 92.4–96.5 s after the launch to provide a background spectrum (Supplementary Fig. 3; BG). In the 97.6–100.2 s after the launch, exactly after the evaporation, a swelling appeared, extending from 11 μm toward longer wavelengths, with a maximum at 12–13 μm (Fig. 2a). At 100.2–103.0 s after the launch, the IR spectra discontinuously fluctuated with time, because hot fragments of the evaporation source drifted into the optical path (Supplementary Fig. 4). At 103.0 s after launch, the IR spectra stabilized (Fig. 2b) and intensified, retaining the shape of the spectra measured 97.6–100.2 s after the launch. Approximately 2.7 s after the evaporation, a sharp absorption at 13.55 ± 0.05 μm began to intensify (Fig. 2c shows the spectrum at 105.6–109.7 s after the launch). This absorption continued for several tens of seconds without showing any change in the band shape (Fig. 2d). The FWHM of the sharp 13.55 ± 0.05 μm band was 0.55 ± 0.05 μm after subtraction of the baseline. This is the first experimental evidence that the IR band arising from Al_2_O_3_ nanoparticles has a bandwidth consistent with theoretical predictions (Supplementary Figs. 5–9) and the unidentified 13 μm band observed around oxygen-rich AGB stars (Fig. 3b).

## Discussion

The IR spectra of the free-flying nanoparticles can be directly compared with astronomical observations, because the dielectric constant of the surrounding gas is similar to that of vacuum (Fig. 3)^19,21–23^. Nanoparticles formed in microgravitational environments will have a greater degree of homogeneity in terms of their size, shape, and crystallinity, because they nucleate and grow in environments with a low Reynolds number, which prevents fluctuations in local concentrations of the evaporated vapor through turbulences^24^. Such homogeneous environments permit gases to undergo extreme cooling, thereby increasing the supersaturation ratio significantly and permitting the formation of nanoparticles of uniform size and shape. The homogeneity of particles generally reduces the bandwidths of their spectra. The previously reported IR spectra of free-flying particles have shown complex shapes and distributions^16^.

The metastable phases γ-, δ-, and θ-Al_2_O_3_, which have been formed in ground-based experiments, have peaks centered at 12 μm with strong satellite peaks in the range of 10–20 μm showing quite different features than those in the spectrum^16,19,25^. The formation of these metastable phases is inconsistent with the results because of the lacking of 12 μm band. Even the other Al_2_O_3_ polymorphs, such as *κ* and *χ*, which are usually formed in the dehydration process of hydrous precursors, accompanied much broad and complex absorption bands in the whole 10–20 μm range^14,22^. Therefore, the most stable phase, α-Al_2_O_3_, is the only possible candidate to reproduce the IR absorption feature at 13.55 ± 0.05 μm (Supplementary Note 1). We calculated the 13 μm feature assuming the properties (facet (Supplementary Fig. 5), temperature (Supplementary Fig. 6), surface contamination (Supplementary Fig. 7), anisotropy (Supplementary Fig. 8), and coagulation (Supplementary Fig. 9)) of α-Al_2_O_3_ by using the Mie theory and the discrete-dipole approximation (Supplementary Note 2). This difference in the peak position in our spectra from that of the astronomically observed 13 μm band, which typically occurs at 12.5–13.0 μm^2^, is possibly a combination of the result of an edge effect of the particle and contamination on their surface in addition to differences in temperature and/or anisotropy of the α-Al_2_O_3_ nanoparticles (Supplementary Note 3). In our ground-based experiments, the condensed θ-Al_2_O_3_ nanoparticles were faceted with edges and occasionally contaminated with amorphous Al_2_O_3_ and Ta when the Ta filament burned out (Supplementary Note 1)^19^. The α-Al_2_O_3_ nanoparticles produced in the current experiment might have also been faceted with sharp edges, which would shift their peak position toward a longer wavelength (Fig. 3e and Supplementary Fig. 5). This confirms that if the origin of the 13 μm band is the α-Al_2_O_3_, the dust particles should have no thick contamination, have approximately a spherical shape, and should rarely aggregate together to maintain a narrow bandwidth. In contrast to the grown particles in this experiment, natural α-Al_2_O_3_ particles in gas outflows of evolved stars will have experienced evaporation and/or sputtering, including incorporation of other elements, and will consequently have round edges. We believe that the differences in the peak wavelength between the experimental measurements and astronomical observations provide us with additional details regarding the condensation processes in AGB stars.

In this study, a significant delay existed between the typical timescale for gas cooling (~10^−3^ s) after the termination of the evaporation (101.4 s after the launch) and the timescale of 2.7 s for the appearance of the sharp 13.55 ± 0.05 μm band. An Al_2_O_3_ nucleation experiment performed under the same gas atmosphere on the ground resulted in the formation of δ-Al_2_O_3_ with a two-step process. In the first step, liquid-like particles that showed a broad absorption extending from 11 μm to longer wavelengths nucleated from the supersaturated vapor at a temperature considerably below the melting point of bulk Al_2_O_3_. In the second step, the crystallization to Al_2_O_3_ was induced in the liquid-like particles while the nanoparticles were freely flying^19^. The 2.7 s delay observed in this study suggests that the liquid-like particles nucleated from the gas, and then the α-Al_2_O_3_ nucleated in these in a similar manner to that in the ground-based experiment. Later, a broad swell appeared with the sharp 13.55 ± 0.05 μm band. The shape of the broad swell in Fig. 2b is consistent with the presence of noncrystalline Al_2_O_3_^6^. However, the identification is not certain because the absorption covers approximately the complete measurement range. With an increasing surface area-to-volume ratio, a stability crossover from α-Al_2_O_3_ to γ-Al_2_O_3_ and from γ-Al_2_O_3_ to amorphous Al_2_O_3_ occurs for surface areas exceeding 75 and 370 m^2^ g^–1^, respectively, which correspond to the sizes of spheres with diameters of ~20 and ~5 nm, respectively^26,27^. In the atmosphere of AGB stars, the size of newly nucleated Al_2_O_3_ particles should be smaller than these sizes because the process began with the agglomeration of molecules (as in the present experiment).

A neutron diffraction study of an Al_2_O_3_ melt kept in a free-flying state at 2500 K showed that 62% of Al was present as AlO_4_, 24% as AlO_5_, and 2% as AlO_6_^28^. Numerical studies have suggested that a large proportion of Al adopts the AlO_4_ tetrahedral form in the melt^29,30^. The metastable forms of Al_2_O_3_ (γ-Al_2_O_3_, δ-Al_2_O_3_, and θ-Al_2_O_3_), which consist of AlO_4_ tetrahedra^6,31,32^, have (owing to their higher concentration of growth units) a higher chance of nucleation than α-Al_2_O_3_, which is composed exclusively of AlO_6_ octahedra. To overcome the larger activation energy required for the nucleation of α-Al_2_O_3_, a higher temperature might be required. Under microgravity conditions, the liquid-like particles might have remained at higher temperatures for a longer time than those on the ground. At a gas pressure of 4.0 × 10^4^ Pa, the temperature of the nanoparticles is almost identical to that of the surrounding gas^19^. Owing to the reported temperature field around the evaporation source at 2226 ± 22 K under an Ar atmosphere of 4.0 × 10^4^ Pa^20^, the nascent nucleated nanoparticles can retain a temperature of about 1000 K for a few seconds. This relatively long incubation time for the nucleation can induce the formation of α-Al_2_O_3_ within the liquid-like particles under microgravity conditions.

The agreement regarding the width of the 13 μm feature observed in this study increases the evidence for the existence of α-Al_2_O_3_ around AGB stars. We expect that future observations at a higher spatial resolution with the James Webb Space Telescope^33^ will confirm the transition from noncrystalline Al_2_O_3_, which shows a broad feature at 11–18 μm, to α-Al_2_O_3_, which shows a sharp 13 μm feature. The spectral region from 10 to 40 μm corresponds to the vibrational modes of various mineral types (e.g., crystalline olivine, crystalline pyroxene, or magnesium sulfide). Therefore, comparing the IR features obtained by astronomical observations with the experimental dataset is crucial to identify the origin of dust^34^. The future observations with the Space Infrared Telescope for Cosmology and Astrophysics (SPICA)^35^ together with microgravity experiments are expected to provide a better understanding of the material evolution in the universe.

## Methods

### In situ IR measurement system

We have previously developed a technique to perform in situ IR measurements during the nucleation and growth of nanoparticles with a gas evaporation method in ground-based experiments^18,21,22^. By using a similar technique, we studied the IR spectra of free-flying nucleating Al_2_O_3_ nanoparticles in a microgravitational environment. The experimental apparatus had to withstand the shock generated during the launch of the sounding rocket and had to fit into the limited space available inside the rocket. Therefore, we specially designed an experimental apparatus equipped with a dispersive IR spectrometer (Fig. 1 and Supplementary Fig. 1). The IR radiation from a SiC source (IRH-1; Siliconit Corp.) passed through the experimental chamber consisting of a 150-mm-long stainless-steel cylinder with an internal diameter of ~65 mm. The radiation was then reflected by a toroidal mirror and passed through the experimental chamber once more to increase the column density. The windows of the experimental chamber were made of ZnSe with an anti-reflection coating; this material has a high transmittance in the 9–17 μm band. The reflected radiation in the Offner-type spectrometer formed an image of the IR spectrum on the detector array (320 × 240 pixels with a pixel pitch of *δx* = 23.5 μm per pixel) of an IR camera (C200C; Nippon Avionics Co., Ltd.) after the dispersion of the wavelength via the convex grating for >300 pixels in the horizontal direction. The signal intensity of the light at each pixel on the array was accumulated at the 240 pixels on the vertical axis. As a result, a single-beam spectrum was obtained at 10 Hz. The wavelength resolution *R* = *λ*/Δ*λ* was 70−101 (Supplementary Fig. 6) with

1 $$\Delta \lambda = \left\{ {\left( {\delta \lambda \times W} \right)^2 + \left( {\delta \lambda \times \Delta {\mathrm{PSF}}} \right)^2} \right\}^{0.5},$$

where *δλ* is the pixel linear dispersion (0.027 μm per pixel), *W* is the slit width (94 μm, corresponding to four pixels on the array detector), and ΔPSF is the performance of the sensor focus. The value of ΔPSF can be expressed as follows:

2 $$\Delta {\mathrm{PSF}} = 1.22 \times \lambda \times F \times \delta x^{-1},$$

where *F* is the *f*-number (5.5) of the camera system. We measured the IR spectrum of a polystyrene film as a standard by using the dispersive IR spectrometer and a Fourier-transform IR spectrometer (Spectrum 400; PerkinElmer, Waltham, MA). The absolute wavelength was determined using the central wavelength of the FWHM of the strong absorption of the standard polystyrene at 13.72 μm.

### Preparation of the nucleation chamber

The chamber was evacuated to a pressure of <1 × 10^−4^ Pa through a quarter-inch stainless-steel tube (Fig. 1g) connected to a vacuum system composed of a turbomolecular pump (TG50F, 50 L s^−1^; Osaka Vacuum, Ltd.) and a scroll-type dry vacuum pump (DIS-90; ULVAC Kiko Inc., Saito City). Three days before the launch, high-purity O_2_ (99.9%) and Ar (>99.9999%) were injected into the experimental chamber. Initially, the pressure was raised to 2 × 10^3^ Pa with O_2_ gas and then raised to a total pressure of 4.0 × 10^4^ Pa with Ar. The gas pressure was measured with a capacitance manometer (CCMT-1000D; ULVAC KIKO Inc.) at a position just outside the valve (Fig. 1g). After the valve was closed, the gas pressure in the experimental chamber was monitored by using a specially coordinated high-resolution pressure gauge (HAV-60KP-V; Sensez Co., Tokyo) to confirm that the pressure was maintained at the time of the experiment. The accuracy of the pressure measurement was ±90 Pa.

### Evaporation source of alumina

Al metal wire (*ϕ* = 0.1 mm, purity 99.99%; Nilaco Corp.) was coiled around a Ta filament (*ϕ* = 0.2 mm, purity 99.95%; Nilaco Corp.), and the assembly was positioned in the experimental chamber as an evaporation source with its axis aligned to the optical axis. The evaporation source was connected to Cu electrodes (PF-SM6-3KV-10A; Kawaso Texcel Co., Osaka) to permit rapid electrical-resistance heating. The evaporation source was positioned 21 mm below the center of the optical path to reduce emission noise during heating.

### Procedures after the launch

The S-520 30 sounding rocket of the Japan Aerospace Exploration Agency (JAXA), which carried the apparatus to an altitude of 312 km, was launched at 8:00 pm JST on 11 September 2015 from the Uchinoura Space Center, Japan. Approximately 50 s after the launch, the rocket entered microgravity. Then, 90 s after the launch, a dc voltage of 1.0 V was applied to the evaporation source; the voltage was then increased at a constant rate of ~0.5 V s^−1^. The temperature of the evaporation source was measured using a pyrometer (ISQ5-LO; LumaSense Technologies, Inc., USA) and K-type thermocouples with diameters of 0.1 mm (a combination of WF-1/8″PT-0.8-2-T-TK-1000 mm/150 mm (Tecsam Co., Ltd., Hsinchu) and KMT-100-100-050 (ANBE SMT Co., Yokohama)) set at the base of the Ta wire. The electrical-resistance heating led to the Al evaporation, and nanoparticles formed from the cooling of evaporated particles. At 101 s after the launch, the evaporation source burned out (Supplementary Fig. 4). The heating of the evaporation source was confirmed with a CMOS camera (MS-M33WT3; MOSWELL Co., Ltd., Yokohama). The recorded data from the flying rocket were received by the telemetry equipment at the Uchinoura Space Center, Japan.

## Electronic supplementary material

Supplementary Information

Description of Additional Supplementary Files

Supplementary Data 1

Supplementary Data 2

Supplementary Data 3

## Data Availability

The datasets generated during and/or analyzed during the current study are available from the corresponding author on reasonable request.
