# Supplementary material for: Sounding-rocket microgravity experiments on alumina dust
Source: Nat Commun. 2018 Sep 19;9:3820. doi: 10.1038/s41467-018-06359-y (PMC6145898; doi:10.1038/s41467-018-06359-y)
Supplement: Supplementary file 1 — Supplementary Information [file 41467_2018_6359_MOESM1_ESM.pdf]

Supplementary Information

Sounding-rocket microgravity experiments on alumina dust

Shinnosuke Ishizuka[1], Yuki Kimura[1,*], Itsuki Sakon[2], Hiroshi Kimura[3], Tomoya Yamazaki[1], Shinsuke Takeuchi[4], and Yuko Inatomi[4,5]

[1]Institute of Low Temperature Science, Hokkaido University, Hokkaido, Sapporo 060-0819, Japan

[2]Department of Astronomy, School of Science, University of Tokyo, 7-3-1 Hongo, Bunkyo-ku, Tokyo 113-0033, Japan

[3]Planetary Exploration Research Center, Chiba Institute of Technology, Tsudanuma 2-17-1, Narashino, Chiba 275-0016, Japan

[4]Institute of Space and Astronautical Science, Japan Aerospace Exploration Agency, 3-1-1 Yoshinodai, Chuo-ku, Sagamihara, Kanagawa 229-8510, Japan

[5]School of Physical Sciences, SOKENDAI (The Graduate University for Advanced Studies), 3-1-1 Yoshinodai, Chuo-ku, Sagamihara, Kanagawa 252-5210, Japan

Correspondence and requests for materials should be addressed to Y.K. (e-mail: ykimura@lowtem.hokudai.ac.jp)

Supplementary Note 1

The spherical tantalum oxide particles have peak positions at 13.24 μm and 14.27 μm for amorphous and nanocrystalline, respectively, at room temperature (Supplementary Fig. 10), which is close to the peak at 13.55 μm from the experiment, shown in Figure 3c. Nevertheless, tantalum oxide does not become a candidate of the origin of the experimental 13.55 μm feature for the following three reasons, even if tantalum oxide formed separately from alumina. The first reason is that tantalum oxide has a wider band widths. Its FWHM are 1.5 μm and 2.4 μm, respectively, which are three to five times more than that of the obtained 13.55 μm feature. The second reason is that the mass absorption coefficient is smaller. The mass absorption coefficient of alumina is more than six times larger than that of tantalum oxides (Supplementary Fig. 10). Instead, the total amount of tantalum in the experimental chamber is only four times more than aluminum. The vapor pressure of aluminum (~10^4 Pa at 2350 K) is much higher than that of tantalum (10^{-5} Pa at 2350 K) and only a small amount of tantalum evaporates before melting due to the very low vapor pressure (less than 1 Pa), even at the melting point of Ta (3290 K). Breaking of the Ta wire can be confirmed from the video image shown in Supplementary Fig. 4. Consequently, tantalum oxide cannot be a major carrier of the 13.55 μm feature. The third reason is that the probability of the oxidation of Ta is lower. The evaporated aluminum vapor consumed oxygen; therefore, no more oxygen remains for the oxidation of tantalum. For these

reasons, we selected tantalum as an evaporation source and as a contaminant of the alumina surface rather than oxide, based on the calculation of the IR spectra given in Figure 3.

Supplementary Note 2

We demonstrated the dependences of facet (Supplementary Fig. 5), temperature (Supplementary Fig. 6), surface contamination (Supplementary Fig. 7), anisotropy (Supplementary Fig. 8) and coagulation (Supplementary Fig. 9) on the IR spectra of α-Al_2O_3 particles with calculations based on the Mie theory and discrete-dipole approximation (DDA) method.[35] The optical constants of α-Al_2O_3, Ta, Ta_2O_5, and amorphous alumina can be found in references 12, 36, 37, and 38, respectively. Faceting, higher temperatures and surface contamination induce a shift of the 13 μm feature toward longer wavelengths.

Supplementary Note 3

High temperatures affect the peak-wavelength position of the 13 μm band of α-Al_2O_3, shifting it toward a longer wavelength (~0.4 μm at 928 K).[12] Although the temperature of the particles can be elevated by the latent heat of crystallization, they are cooled down immediately by the buffer Ar gas, which can warm up by 500 K through short-term heating. In this study, the temperature of the electrode measured by the thermocouple was 373 K at

110 s after heating up to 673 K at ~104 s. Accordingly, the temperature effect is not sufficient to account for the shift of 0.55 μm. Anisotropic growth would account for the shift only if the α-Al_2O_3 nanoparticles were all flattened in the direction of the *c*-axis in a ratio of 0.4.[13] This is also implausible because the α-Al_2O_3 particles were formed from liquid particles and should therefore have near-spherical shapes.

Supplementary References

1. Zeidler, S., Posch, Th. & Mutschke, H. Optical constants of refractory oxides at high temperatures: Mid-infrared properties of corundum, spinel, and α-quartz, potential carriers of the 13 μm feature. *Astron. Astrophys.* **553,** A81 (2013).
2. Takigawa, A., Tachibana, S., Nagahara, H. & Ozawa, K. Evaporation and condensation kinetics of corundum: The origin of the 13 μm feature of oxygen-rich AGB stars. *Astrophys. J., Suppl. Ser.* **218,** 2 (2015).
3. Draine, B. T. & Flatau, P. J. Discrete-dipole approximation for scattering calculations: *J. Opt. Soc. Am. A* **11,** 1491 (1994).
4. Ordal, M., Bell, R. J., Alexander, R. W., Jr, Newquist, L. A. & Querry, M. R. Optical properties of Al, Fe, Ti, Ta, W, and Mo at submillimeter wavelengths. *Applied Optics* **27,** 1203 (1988).

5. Bright, T. J. *et al*. Infrared optical properties of amorphous and nanocrystalline Ta_2O_5 thin films. *J. Appl. Phys.* **114,** 083515 (2013).

6. Begemann, B. *et al*. Aluminum oxide and the opacity of oxygen-rich circumstellar dust in the 12-17 micron range. *Astrophys. J.* **476,** 199–208 (1997).

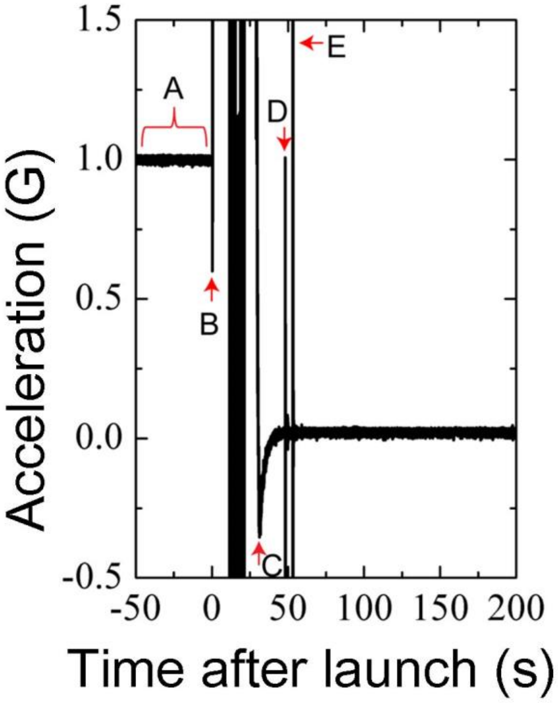

Supplementary Fig. 1. Gravitational acceleration of the sounding rocket measured during the experiment. Labels A to E indicate the following events: (**A)** before launch; (**B**) ignition; (**C**) termination of ignition; (**D**) despinning by spreading of yo-yo; (**E**) opening of nose cone for heat release. The gravitational acceleration was measured with a triaxial analogue accelerometer module (Model 2470-002; Silicon Designs Inc., Kirkland, WA), positioned on a baseplate just above the experimental system.

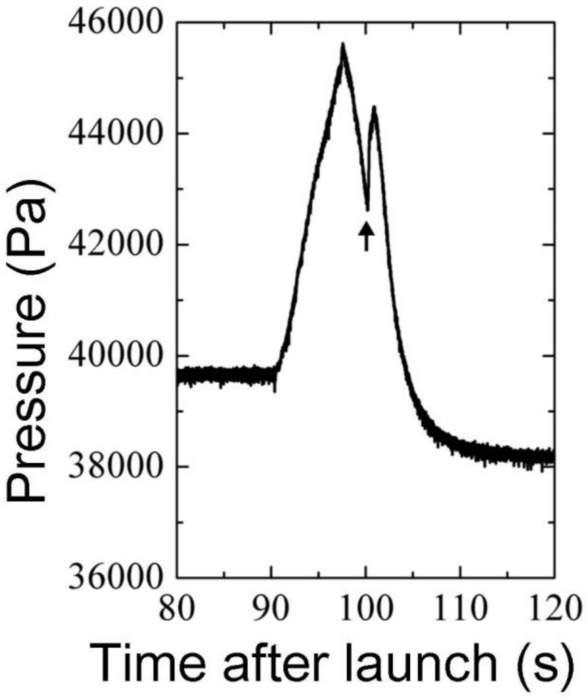

Supplementary Fig. 2. Pressure in the experimental chamber measured during the experiment. Owing to the heated evaporation source, the pressure began to increase from 39700 Pa 90 s after launch. The pressure started to decrease once the evaporation source had burned out, as indicated by the arrow. At 110 s after launch, the pressure reached approximately 38000 Pa, indicating that 1700 Pa of O_2 had been consumed in the oxidation of the evaporated Al. It was confirmed in an Al-free experiment that the oxidation of Ta has a negligible effect on the oxygen consumption.

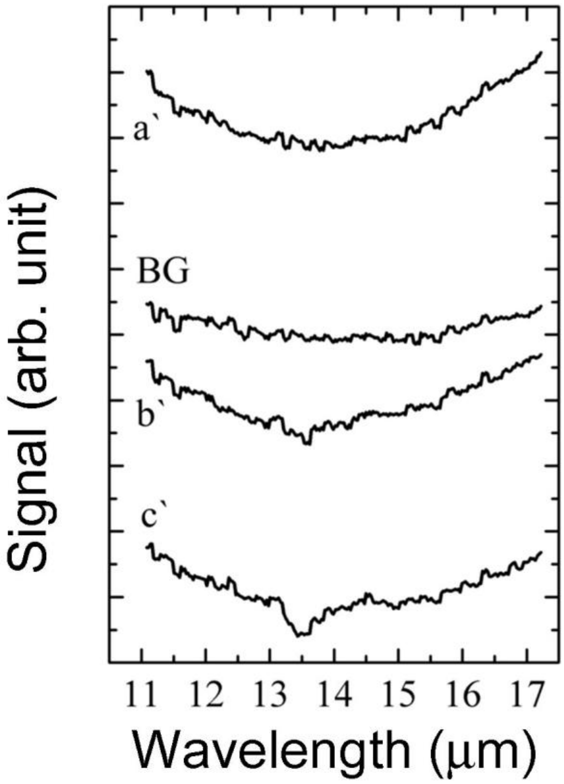

Supplementary Fig. 3. Accumulated raw-signal IR spectra: BG: 92.4–96.5 s after launch; **a′**: 97.6–100.2 s after launch; **b′**: 103.0–104.5 s after launch; **c′**: 105.6–109.7 s after launch. The spectra were not offset, and the absorption appeared as a reduction in the signal. After the subtraction of the spectrum measured when the IR light was blinded (F_{bl}), the absorbance spectra were obtained from the sample spectra (F) and background spectra (F_{BG});

$$A = -\ln\left(\frac{F - F_{bl}}{F_{BG} - F_{bl}}\right).$$

The corresponding absorbance spectra when BG was used as a background spectrum for a′–c′ are shown as a–c in Figure 2.

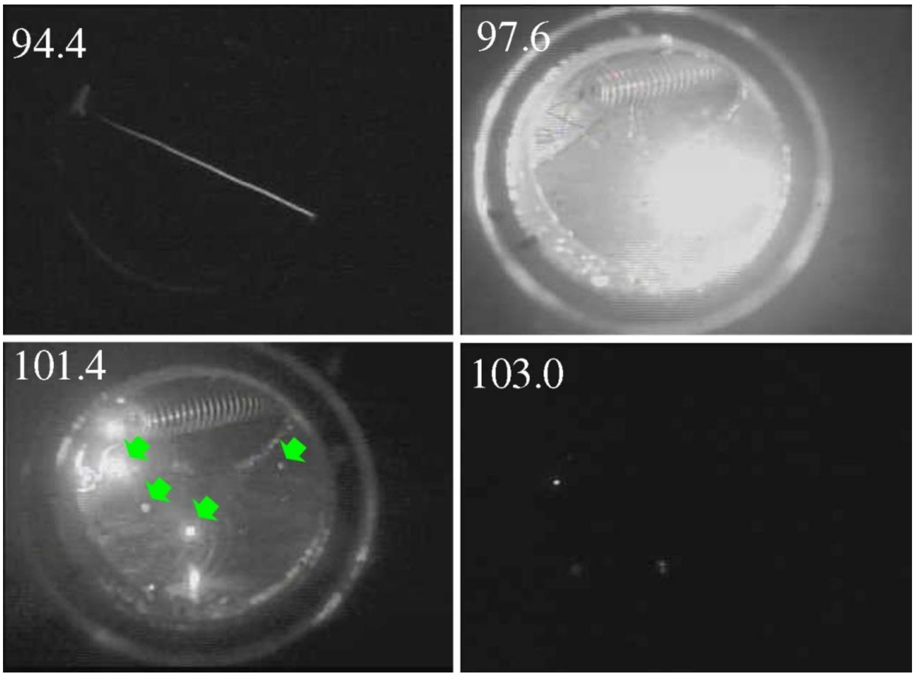

Supplementary Fig. 4. Still photographs of the evaporation source in the experimental chamber. The number in each image presents the time in seconds after the launch. During the observation of the background spectra, the temperature of the evaporation source (<1200 K) was well below the evaporation temperature of Al, as shown at 94.4 s. The equilibrium vapor pressure of Al at 1357 K is 0.1 Pa. The significant brightness of the evaporation source at 97.6 s, which corresponds to the spectrum in Figure 2a, suggests that the evaporation of Al occurred. Fragments of the burned-out Ta filament with an Al wire are indicated by the arrows at 101.4 s. The last panel confirms that the evaporation was complete when spectra b and c in Figure 2 were recorded.

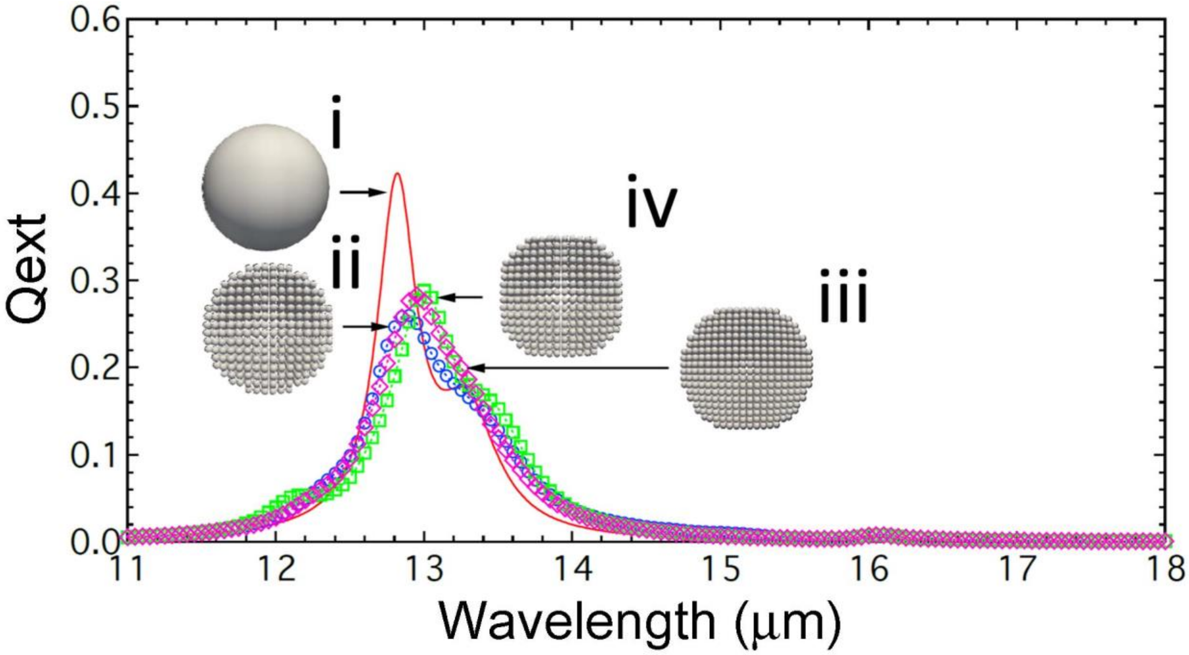

Supplementary Fig. 5. Shape (facet) dependences of calculated IR spectra of α-Al_2O_3 particles at 551 K: (**i**) sphere according to Mie theory, (**ii**) sphere; (**iii**) sphere truncated at the top and bottom; (**iv**) faceted particle using DDA method.

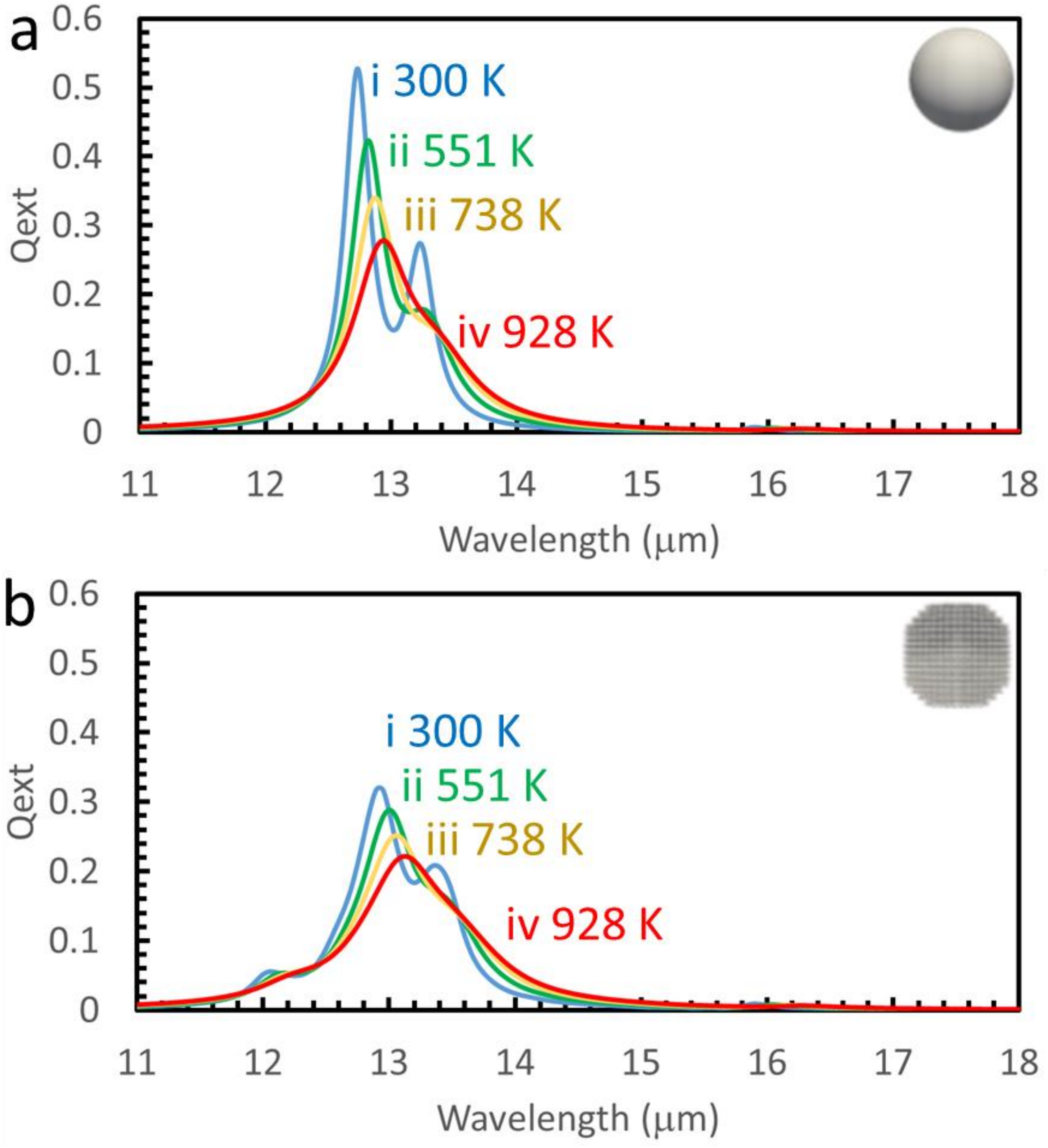

Supplementary Fig. 6. Temperature dependences of calculated IR spectra of α-Al_2O_3 particles: (**a**) sphere calculated with Mie theory; (**b**) faceted particle calculated using DDA method. Spectra **i-iv** were recorded at a temperatures of 300, 551, 738, and 928 K, respectively.

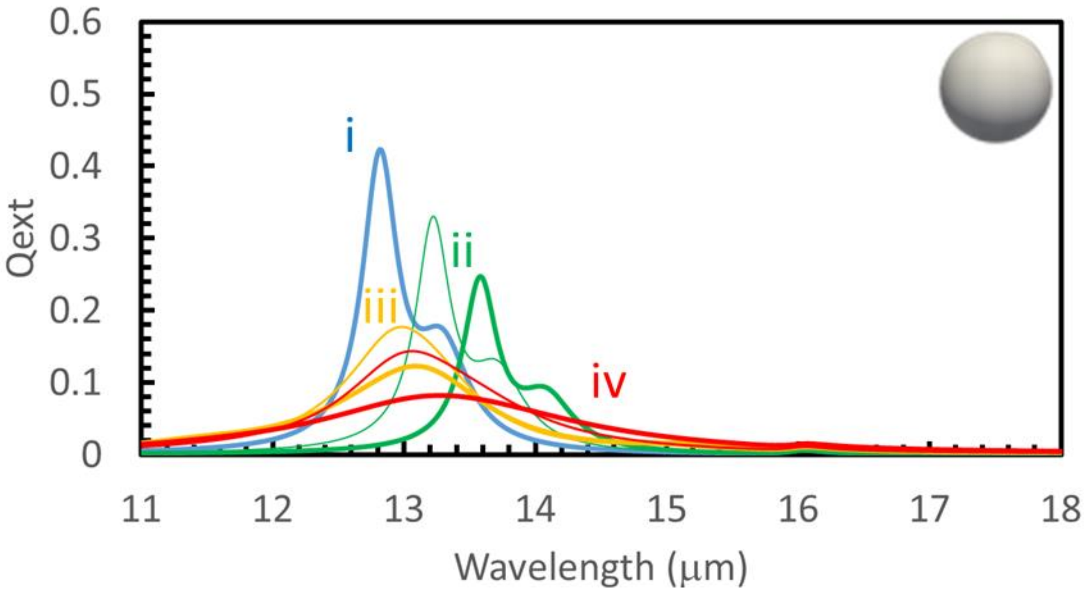

Supplementary Fig. 7. Thickness dependences of surface contamination of calculated IR spectra of spherical α-Al_2O_3 particles at 551 K: (**i**) no contamination; (**ii**–**iv**) contaminated by Ta, amorphous Ta_2O_5 and amorphous alumina, respectively. Thin and thick curves indicate the thicknesses of the surface contamination of 2.5 nm and 5 nm, respectively. The optical constants for Ta, Ta_2O_5, and porous amorphous alumina are referred from references 36, 37 and 38, respectively.

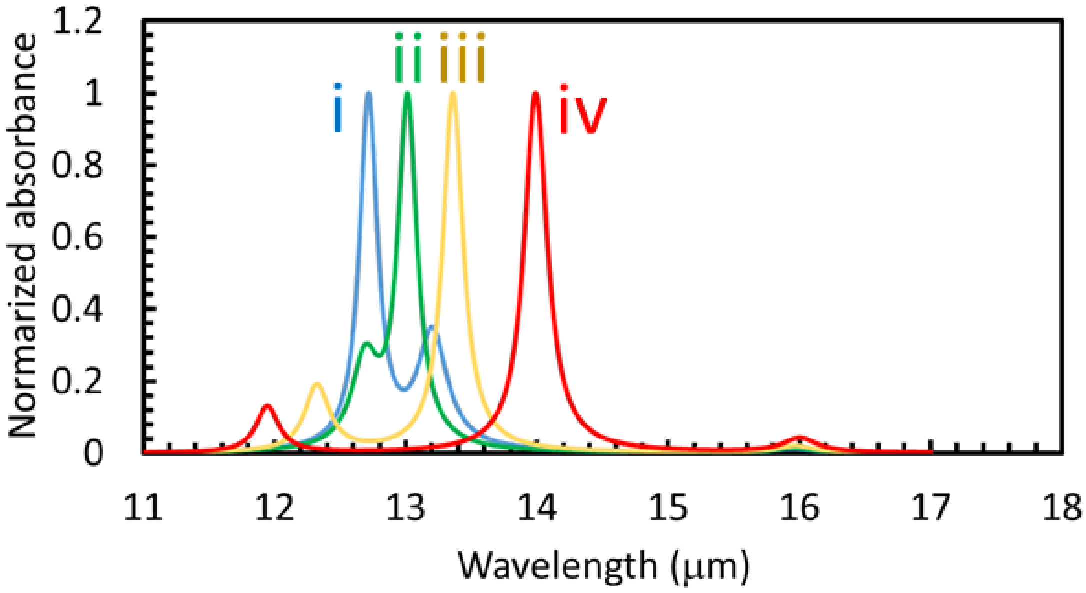

Supplementary Fig. 8. Anisotropy dependence of IR spectra, calculated with Mie theory, of α-Al_2O_3 particles at 300 K: (**i**) spheres and (**ii**–**iv**) oblates flattened along the *c*-axis with ratios of 0.7, 0.5, and 0.3, respectively.[13]

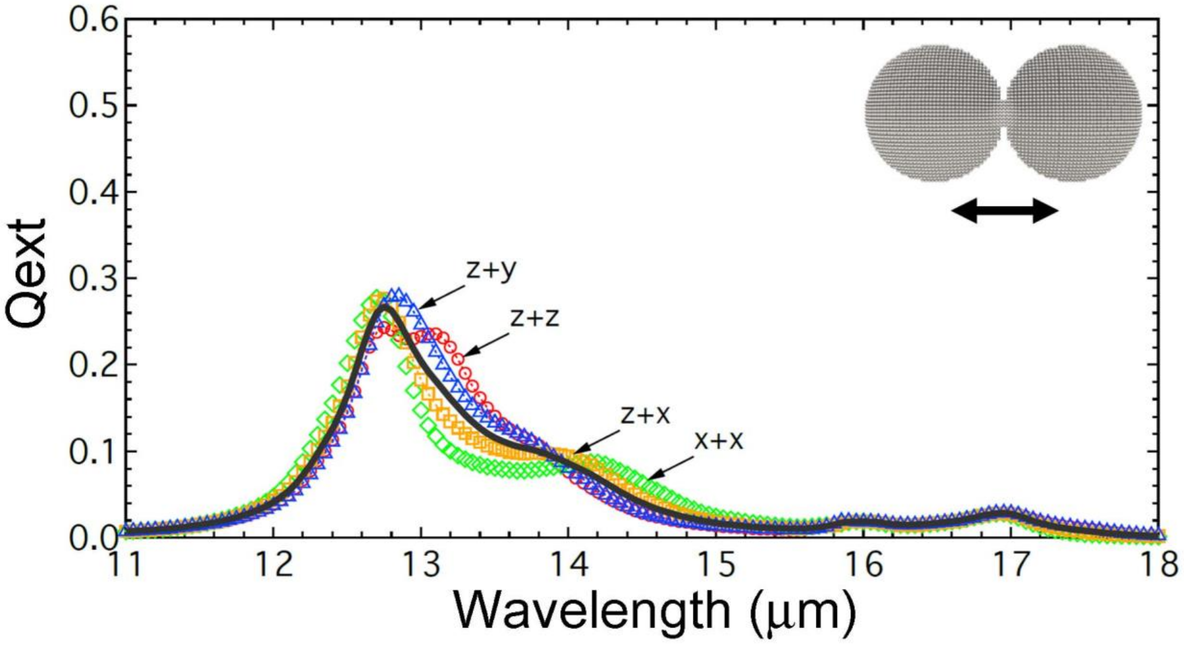

Supplementary Fig. 9. Calculated IR spectra of two connected α-Al_2O_3 particles at 551 K using DDA method. x + x, z + x, z + y and z + z are the crystallographic orientation of each particle in respect to the direction shown by the arrow.

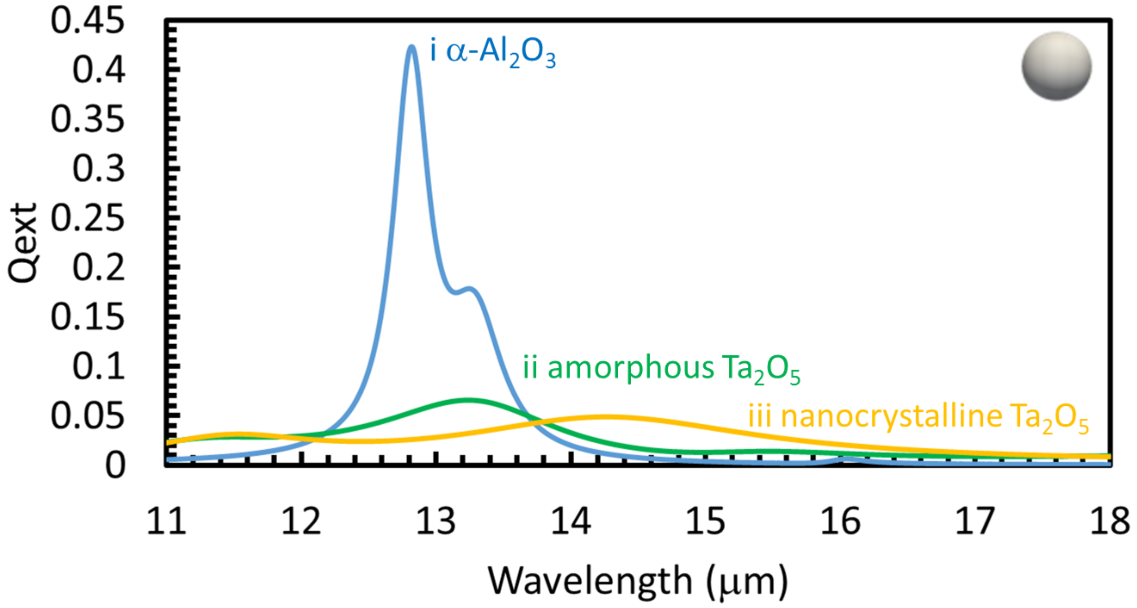

Supplementary Fig. 10. The calculated IR spectra of the sphere shape using the Mie theory. (i) α-Al_2O_3 particles at 551 K; (ii) amorphous Ta_2O_5 at room temperature and (iii) nanocrystalline Ta_2O_5 at room temperature. The optical constant for Ta_2O_5 is referred from reference 37.

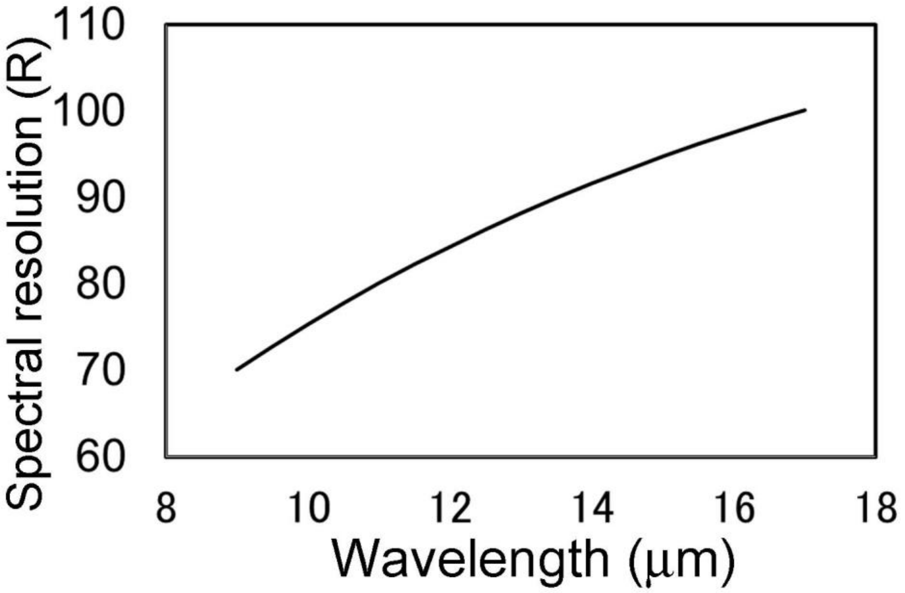

Supplementary Fig. 11. Relationship between the spectral resolution and the wavelength for the optical system in Figure 1.
