# Supplementary material for: Sounding-rocket microgravity experiments on alumina dust
Source: Nat Commun. 2018 Sep 19;9:3820. doi: 10.1038/s41467-018-06359-y (PMC6145898; doi:10.1038/s41467-018-06359-y)
Supplement: Supplementary file 2 — Description of Additional Supplementary Files [file 41467_2018_6359_MOESM2_ESM.pdf]

Description of Additional Supplementary Files

File Name: Supplementary Data 1
Description: list of optical constants and efficiency coefficients for extinction calculated using the Mie theory.

File Name: Supplementary Data 2
Description: list of efficiency coefficients for extinction calculated using DDA method.

File Name: Supplementary Data 3
Description: list of particle conditions for IR calculation corresponding to the data in two excel files. Aspect ratio of ellipsoid particle is 0.9 for the data name of Qext551_N30192 and Qext551_N30192_Ta.
