# Supplementary material for: Sounding-rocket microgravity experiments on alumina dust
Source: Nat Commun. 2018 Sep 19;9:3820. doi: 10.1038/s41467-018-06359-y (PMC6145898; doi:10.1038/s41467-018-06359-y)
Supplement: Supplementary file 5 — Supplementary Data 3 [file 41467_2018_6359_MOESM5_ESM.docx]

| Data name | Temperature (K) | Shape | Surface contamination | Number of dipole |
| --- | --- | --- | --- | --- |
| Qext300_N2176 | 300 | 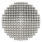  Sphere | - | 2176 |
| Qext300_N2920 | 300 | 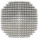  Facet | - | 2920 |
| Qext551_N2176 | 551 | 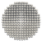  Sphere | - | 2176 |
| Qext551_N2176_Ta | 551 | 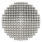  Sphere | Ta  3.125 nm | 2176 |
| Qext551_N2920 | 551 | 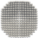  Facet | - | 2920 |
| Qext551_N2920_Ta | 551 | 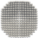  Facet | Ta  3.125 nm | 2920 |
| Qext551_N4160 | 551 | 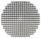  Truncated | - | 4160 |
| Qext551_N30192 | 551 | 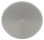  Ellipsoid | - | 30192 |
| Qext551_N30192_Ta | 551 | 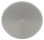  Ellipsoid | Ta  2.33-2.59 nm | 30192 |
| Qext551_N33552 | 551 | 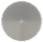  Sphere | - | 33592 |
| Qext551_N33552_Ta | 551 | 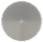  Sphere | Ta  1.25 nm | 33592 |
| Qext551_N33552_Ta2 | 551 | 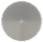  Sphere | Ta  2.5 nm | 33592 |
| Qext551_N47408 | 551 | 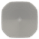  Facet | - | 47408 |
| Qext551_N47408_Ta | 551 | 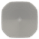  Facet | Ta  1.25 nm | 47408 |
| Qext551_N67104avg | 551 | 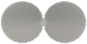 | - | 67104 |
| Qext551_N67104xx | 551 | 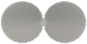 | - | 67104 |
| Qext551_N67104zx | 551 | 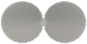 | - | 67104 |
| Qext551_N67104zy | 551 | 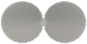 | - | 67104 |
| Qext551_N67104zz | 551 | 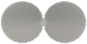 | - | 67104 |
| Qext551_N137376 | 551 | 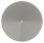  Sphere | - | 137376 |
| Qext738_N2176 | 738 | 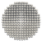  Sphere | - | 2176 |
| Qext738_N2920 | 738 | 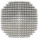  Facet | - | 2920 |
| Qext928_N2176 | 928 | 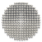  Sphere | - | 2176 |
| Qext928_N2920 | 928 | 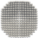  Facet | - | 2920 |
| Qext300para_sphere | 300 | 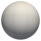  Sphere | - | - |
| Qext300perp_sphere | 300 | 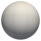 | - | - |
| Qext300_sphere | 300 | 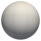 | - | - |
| Qext551para_sphere | 551 | 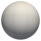 | - | - |
| Qext551para_sphere10Al2O3c | 551 | 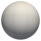 | compact a-Al2O3  2.5 nm | - |
| Qext551para_sphere10Al2O3p | 551 | 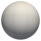 | porous a-Al2O3  2.5 nm | - |
| Qext551para_sphere10Ta | 551 | 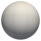 | Ta  2.5 nm | - |
| Qext551para_sphere10Ta2O5 | 551 | 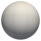 | Ta2O5  2.5 nm | - |
| Qext551para_sphere20Al2O3c | 551 | 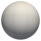 | compact a-Al2O3  5.0 nm | - |
| Qext551para_sphere20Al2O3p | 551 | 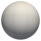 | porous a-Al2O3  5.0 nm | - |
| Qext551para_sphere20Ta | 551 | 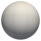 | Ta  5.0 nm | - |
| Qext551para_sphere20Ta2O5 | 551 | 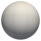 | Ta2O5  5.0 nm | - |
| Qext551perp_sphere | 551 | 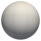 | - | - |
| Qext551perp_sphere10Al2O3c | 551 | 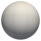 | compact a-Al2O3  2.5 nm | - |
| Qext551perp_sphere10Al2O3p | 551 | 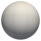 | porous a-Al2O3  2.5 nm | - |
| Qext551perp_sphere10Ta | 551 | 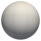 | Ta  2.5 nm | - |
| Qext551perp_sphere10Ta2O5 | 551 | 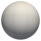 | Ta2O5  2.5 nm | - |
| Qext551perp_sphere20Al2O3c | 551 | 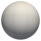 | compacta-Al2O3  5.0 nm | - |
| Qext551perp_sphere20Al2O3p | 551 | 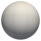 | porous a-Al2O3  5.0 nm | - |
| Qext551perp_sphere20Ta | 551 | 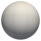 | Ta  5.0 nm | - |
| Qext551perp_sphere20Ta2O5 | 551 | 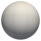 | Ta2O5  5.0 nm | - |
| Qext551_sphere | 551 | 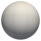 | - | - |
| Qext551_sphere10Al2O3c | 551 | 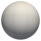 | compact a-Al2O3  2.5 nm | - |
| Qext551_sphere10Al2O3p | 551 | 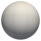 | porous a-Al2O3  2.5 nm | - |
| Qext551_sphere10Ta | 551 | 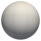 | Ta  2.5 nm | - |
| Qext551_sphere10Ta2O5 | 551 | 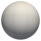 | Ta2O5  2.5 nm | - |
| Qext551_sphere20Al2O3c | 551 | 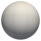 | compact a-Al2O3  5.0 nm | - |
| Qext551_sphere20Al2O3p | 551 | 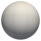 | porous a-Al2O3  5.0 nm | - |
| Qext551_sphere20Ta | 551 | 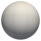 | Ta  5.0 nm | - |
| Qext551_sphere20Ta2O5 | 551 | 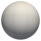 | Ta2O5  5.0 nm | - |
| Qext738para_sphere | 738 | 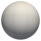 | - | - |
| Qext738perp_sphere | 738 | 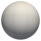 | - | - |
| Qext738_sphere | 738 | 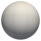 | - | - |
| Qext928para_sphere | 928 | 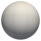 | - | - |
| Qext928perp_sphere | 928 | 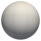 | - | - |
| Qext928_sphere | 928 | 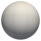 | - | - |
| Amorphous_Ta2O5 | 300 | 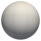 | - | - |
| Nanocrystalline_Ta2O5 | 300 | 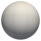 | - | - |
